# Generative AI intervention clinical trials: a call for pre-registration – correspondence

**DOI:** 10.1097/JS9.0000000000001690

**Published:** 2024-05-22

**Authors:** Haiyang Wu, Zaijie Sun, Qiang Guo, Xiaojun Liu, Kunming Cheng, Cheng Li

**Affiliations:** a Department of Orthopaedics, The First Affiliated Hospital of Zhengzhou University; b Department of Intensive Care Unit, The Second Affiliated Hospital of Zhengzhou University, Zhengzhou; c Department of Orthopaedics, Xiangyang Central Hospital, Affiliated Hospital of Hubei University of Arts and Science, Xiangyang; d Department of Orthopedics, Baodi Clinical College of Tianjin Medical University; e Department of Clinical College of Neurology, Neurosurgery and Neurorehabilitation, Tianjin Medical University, Tianjin; f Department of Spine Surgery, Wangjing Hospital, China Academy of Chinese Medical Sciences, Beijing, China; g Center for Musculoskeletal Surgery (CMSC), Charité-Universitätsmedizin Berlin, Corporate Member of Freie Universität Berlin, Humboldt University of Berlin, Berlin Institute of Health, Berlin, Germany

*Dear Editor,*

Recently, *International Journal of Surgery* has published clinical trials on generative artificial intelligence (AI).^1^ Clinical trials represent public events with direct ramifications on medical practice and patient well-being. However, a dearth of transparency and information disclosure could precipitate misdirection and hazards. In pursuit of upholding integrity and equity in medical research, the International Committee of Medical Journal Editors (ICMJE) issued a declaration in 2004 mandating that member journals, effective 1 July 2005, exclusively accept trial results registered with public clinical trial registries and require the registration number upon publication. This edict’s implementation propelled clinical trial registration into a requisite procedure. Presently, numerous clinical trial registration platforms exist, including the largest clinicaltrials.gov. Clinical trial registration empowers the public to independently access pertinent information such as ongoing trials, recruitment status, research designs, and anticipated outcomes, thereby enhancing research transparency and traceability. Simultaneously, registration protocols aid in thwarting selective reporting and outcome manipulation, thereby safeguarding the objectivity and credibility of research findings. Consequently, the establishment and enforcement of clinical trial registration systems hold paramount significance in upholding the ethical and scientific standards of medical research and safeguarding patient rights.

The advent of generative AI, employing diverse machine learning methods to learn patterns from data and create entirely original text, images, and even videos, marks a pivotal moment in artificial intelligence. The year 2022 was hailed as the ‘Year of Generative AI,’ with models like OpenAI’s ChatGPT representing a focal point in the AI landscape. In recent years, an increasing number of studies have incorporated generative AI into clinical research. Notably, a study published in *Science* showcased results from a clinical trial involving 453 highly educated professionals, demonstrating that the use of ChatGPT significantly enhanced productivity, improved task quality, and reduced task completion time^2^. To our knowledge, this is the first ChatGPT-related clinical trial with the complete registration number.

Since then, an array of clinical trials evaluating ChatGPT’s applications in oncology, radiology, surgery, and critical care medicine has emerged, contributing to a burgeoning body of research^3-5^. However, amidst these published clinical studies, a significant proportion has overlooked a crucial aspect of clinical trials: registration. A search on clinicaltrials.gov using ‘ChatGPT’ as a keyword yields only a dozen registered clinical studies, a stark mismatch compared to the number of published ChatGPT-related clinical trials. This discrepancy underscores the prevalent neglect of clinical registration in the current landscape of published trials.

Given the nascent nature of generative AI, substantial controversy persists within the scientific community regarding its principles and applications. Consequently, delineating which generative AI clinical trials necessitate registration proves challenging. In many of the currently published clinical studies, researchers utilize generative AI, such as ChatGPT, as a control group, comparing its efficacy with that of clinical professionals in diagnosis, treatment, and even prognosis prediction. The classification of these studies as quality improvement endeavors or clinical trials remains subject to further delineation. However, based on our interpretation of the ICMJE guidelines, all generative AI clinical trials involving human data should undergo prospective registration to ensure trial transparency and safety.

In light of the transformative potential of generative AI in healthcare, proactive measures are warranted to align research practices with ethical and scientific standards. As such, concerted efforts are needed to foster a culture of responsible conduct and accountability within the research community. By advocating for the registration of generative AI clinical trials, we could collectively advance the field toward greater transparency, rigor, and, ultimately, improved patient outcomes.

## Ethical approval

This study does not include any individual-level data and thus does not require any ethical approval.

## Sources of funding

This study is supported by China Postdoctoral Science Foundation (2022M720385) and Beijing JST Research Funding (YGQ-202313).

## Author contribution

H.W.: conceptualization, methodology, data curation, formal analysis, resources, investigation, and writing – original draft; Z.S. and Q.G.: conceptualization, methodology, data curation, formal analysis, resources, and investigation; X.L. and K.C.: conceptualization and methodology; C.L.: methodology, data curation, formal analysis, resources, investigation, and writing – review and editing.

## Conflicts of interest disclosure

The authors declare no conflicts of interest.

## Research registration unique identifying number (UIN)

1. Name of the registry: not applicable.
2. Unique identifying number or registration ID: not applicable.
3. Hyperlink to your specific registration (must be publicly accessible and will be checked): not applicable.

## Guarantor

Haiyang Wu and Cheng Li.

## Data availability statement

The data underlying this article will be shared by the corresponding author on reasonable request.
